# Status of HIV and hepatitis C virus infections among prisoners in the Middle East and North Africa: review and synthesis

**DOI:** 10.7448/IAS.19.1.20873

**Published:** 2016-05-27

**Authors:** Marieke Heijnen, Ghina R Mumtaz, Laith J Abu-Raddad

**Affiliations:** 1 Infectious Disease Epidemiology Group, Weill Cornell Medicine – Qatar, Cornell University, Qatar Foundation – Education City, Doha, Qatar; 2 Department of Healthcare Policy and Research, Weill Cornell Medicine, Cornell University, New York, NY, USA; 3 Department of Infectious Disease Epidemiology, Faculty of Epidemiology and Population Health, London School of Hygiene and Tropical Medicine, London, UK; 4 College of Public Health, Hamad bin Khalifa University, Doha, Qatar

**Keywords:** HIV, HCV, incarceration, Middle East and North Africa, prisons, PWID

## Abstract

**Introduction:**

The status of HIV and hepatitis C virus (HCV) infections among incarcerated populations in the Middle East and North Africa (MENA) and the links between prisons and the HIV epidemic are poorly understood. This review synthesized available HIV and HCV data in prisons in MENA and highlighted opportunities for action.

**Methods:**

The review was based on data generated through the systematic searches of the MENA HIV/AIDS Epidemiology Synthesis Project (2003 to December 15, 2015) and the MENA HCV Epidemiology Synthesis Project (2011 to December 15, 2015). Sources of data included peer-reviewed publications and country-level reports and databases.

**Results and discussion:**

We estimated a population of 496,000 prisoners in MENA, with drug-related offences being a major cause for incarceration. Twenty countries had data on HIV among incarcerated populations with a median prevalence of 0.6% in Afghanistan, 6.1% in Djibouti, 0.01% in Egypt, 2.5% in Iran, 0% in Iraq, 0.1% in Jordan, 0.05% in Kuwait, 0.7% in Lebanon, 18.0% in Libya, 0.7% in Morocco, 0.3% in Oman, 1.1% in Pakistan, 0% in Palestine, 1.2% in Saudi Arabia, 0% in Somalia, 5.3% in Sudan and South Sudan, 0.04% in Syria, 0.05% in Tunisia, and 3.5% in Yemen. Seven countries had data on HCV, with a median prevalence of 1.7% in Afghanistan, 23.6% in Egypt, 28.1% in Lebanon, 15.6% in Pakistan, and 37.8% in Iran. Syria and Libya had only one HCV prevalence measure each at 1.5% and 23.7%, respectively. There was strong evidence for injecting drug use and the use of non-sterile injecting-equipment in prisons. Incarceration and injecting drugs, use of non-sterile injecting-equipment, and tattooing in prisons were found to be independent risk factors for HIV or HCV infections. High levels of sexual risk behaviour, tattooing and use of non-sterile razors among prisoners were documented.

**Conclusions:**

Prisons play an important role in HIV and HCV dynamics in MENA and have facilitated the emergence of large HIV epidemics in at least two countries, Iran and Pakistan. There is evidence for substantial but variable HIV and HCV prevalence, as well as risk behaviour including injecting drug use and unprotected sex among prisoners across countries. These findings highlight the need for comprehensive harm-reduction strategies in prisons.

## Introduction

HIV and hepatitis C virus (HCV) infections in prisons have been identified globally as a public health problem [1]. High HIV prevalence among prisoners has been documented in multiple countries, with a prevalence of at least 10% in 18 low-income countries [2]. High HCV prevalence among prisoners has also been reported across countries with one in four prisoners globally testing HCV positive [3]. Although HIV and HCV transmission in prisons is difficult to investigate in epidemiological studies [2], there has been evidence of prisons playing a role in the emergence and amplification of HIV epidemics [4]. Prisons have characteristics that can increase the risk of HIV and HCV exposure, including high rates of injecting drug use, often with non-sterile injecting equipment, unprotected sex and use of non-sterile tattooing equipment or razors for shaving [5,6]. With this context, the recently launched Joint United Nations Programme on HIV/AIDS (UNAIDS) Strategy for 2016 to 2021, titled “On the Fast-Track to end AIDS,” has highlighted, for the first time, a specific global target for HIV response among incarcerated populations (Target 6) [7].

People who inject drugs (PWID), one of the key populations at risk of HIV and HCV infections, are often overrepresented among prison inmates; and imprisonment is a common and recurring event for many PWID [8,9]. Incarceration of PWID, who often continue to inject drugs in prison, together with other risk behaviours, is conducive for a favourable environment for the transmission of HIV and HCV in prisons.

Injecting drug use is an important transmission route for HIV and HCV in the Middle East and North Africa (MENA) [10,11]. This region has several vulnerability factors for the practice; for example, the majority (80%) of the global supply of heroin is produced in Afghanistan [12], and over three-quarters of this is trafficked through Iran and Pakistan [13]. Increased purity and availability of heroin in MENA at lower cost may have contributed to higher injecting drug use [14]. Recently, emerging HIV epidemics have been documented among PWID [10] and men who have sex with men (MSM) [15] in different MENA countries. The HIV epidemic in MENA is one of the two fastest growing HIV epidemics worldwide with most country-specific epidemics emerging only in the last decade [16]. MENA also appears to have the highest HCV prevalence in the population at large in the world [17,18]. The highest HCV prevalence worldwide is found in Egypt at 14.7% [19,20], followed by Pakistan with one of the largest HCV-infected populations globally with a national HCV prevalence of 4.8% [21,22].

Against this global background and regional context of emerging HIV epidemics in MENA, it is important to assess HIV and HCV infection levels among prisoners in this region and establish the role that prisons play in the transmission dynamics. The aim of this review is to synthesize available data on HIV and HCV prevalence among prisoners, assess risk behaviours and risk factors in prisons, and delineate the role of incarceration in the HIV epidemic dynamics in MENA. Our overarching goal is to provide a coherent overview of the status of these infections in prisons that can highlight opportunities for action and inform resource allocation as well as HIV and HCV policy and programming priorities.

## Methods

This narrative review is based on a compilation and synthesis of data that relate to HIV and HCV infections and prisons in MENA. The data have been identified through comprehensive systematic searches of literature, conducted as part of the MENA HIV/AIDS Epidemiology Synthesis Project [11,23] and the MENA HCV Epidemiology Synthesis Project [24]. Data collection in the MENA HIV/AIDS Epidemiology Synthesis Project started in mid-2003 with the mandate to collect and synthesize all available data on HIV, sexually transmitted infections, and sexual behaviour in MENA through a series of systematic sub-studies focused on different aspects of the HIV epidemic [10,11,15,23,25–28]. Data collection on the MENA HCV Epidemiology Synthesis Project started in April 2011 with the mandate to characterize HCV epidemiology in MENA and estimate country-specific population-level HCV prevalence through a series of systematic sub-studies focused on sub-regional analyses [20,24,29–36]. No publication date or language limitations were set for either of these projects, and thus, they include all identified and available data for the region published at any time. As a consequence of these projects, a substantial volume of data on HIV and HCV infections and prisons has been identified that warranted the conduct of the present study. Nearly all these data were generated and published after 1990, given the relatively recent discovery of both viruses (in the 1980s), but most identified and included data in our review were generated and published after 2000 with the strong growth in HIV and HCV research in MENA following increased funding for HIV research thanks to international donors [37] and the growing interest in HCV infection such as after the discovery of the large HCV epidemic in Egypt [20].

Data sources for our study included peer-reviewed publications identified through PubMed and Embase searches and publications in local and regional journals not indexed in PubMed or Embase but identified through specific custom searches (without any language or date restrictions). The search terms used in the literature searches included broad geographic, infection, and epidemiological qualifiers to ensure inclusiveness, as can be seen in different sub-studies of the two Synthesis Projects [10,11,15,20,23–26,28–38].

A substantial volume of relevant unpublished country-level reports and databases were also identified through the MENA HIV/AIDS Epidemiology Synthesis Project database [11,23]. Twenty-four countries were included in our definition of MENA: Afghanistan, Algeria, Bahrain, Djibouti, Egypt, Iran, Iraq, Jordan, Kuwait, Lebanon, Libya, Morocco, Oman, Pakistan, Palestine, Qatar, Saudi Arabia, Somalia, Sudan, South Sudan, Syria, Tunisia, United Arab Emirates (UAE), and Yemen. Because of the recent independence of South Sudan, Sudan is mainly referred to as one country in this review, including Sudan and South Sudan, unless the data allows otherwise.

Country-level data extracted from studies included HIV and HCV prevalence and incidence among prisoners, prevalence of injecting drug use and use of non-sterile injected equipment in prisons, and prevalence of sexual and other risk behaviours among prisoners. We also extracted and summarized measures of association from studies that investigated history of imprisonment and history of injecting drug use among prisoners as risk factors for HIV or HCV infections. Imprisonment rates were extracted from the International Centre for Prison Studies [39] and complemented with additional data on country-specific imprisonment rates from included publications, where applicable. A map of the region displaying imprisonment rates was developed using Tableau software (Version 9.2, Seattle, WA, USA). In text and tables, the numbers of decimal points in all included percentages were as per the original source, up to one decimal place. Percentages reported with more than one decimal place have been rounded to one decimal place. The only exception to this rule are HIV and HCV prevalence measures below 0.1%, in which case two decimal places are provided.

The above data were synthesized using a triangulation approach [40] to infer key features of HIV and HCV epidemiology as they relate to incarceration. This approach consists of an integrated analysis of multiple sources of data examined side-by-side to draw a comprehensive picture of HIV and HCV epidemiology and prisons. We estimated the prisoner population by weighing country-level imprisonment rates by adult population size, as extracted from the United Nations Population Division Database [41]. When more than one prisoner population estimate was available per country, the median of estimates was used. We described HIV and HCV prevalence among prisoners in each study included in the review and summarized them at country-level using the median and total range of values. In addition, we provided an overall estimate (median) of HIV/HCV prevalence in the region and accompanying interquartile range (IQR). All odds ratios (ORs) included were extracted from multivariable analyses with adjustment of confounders, unless otherwise specified.

## Results

### Imprisonment and drug use

#### Imprisonment rates

**Figure 1:**
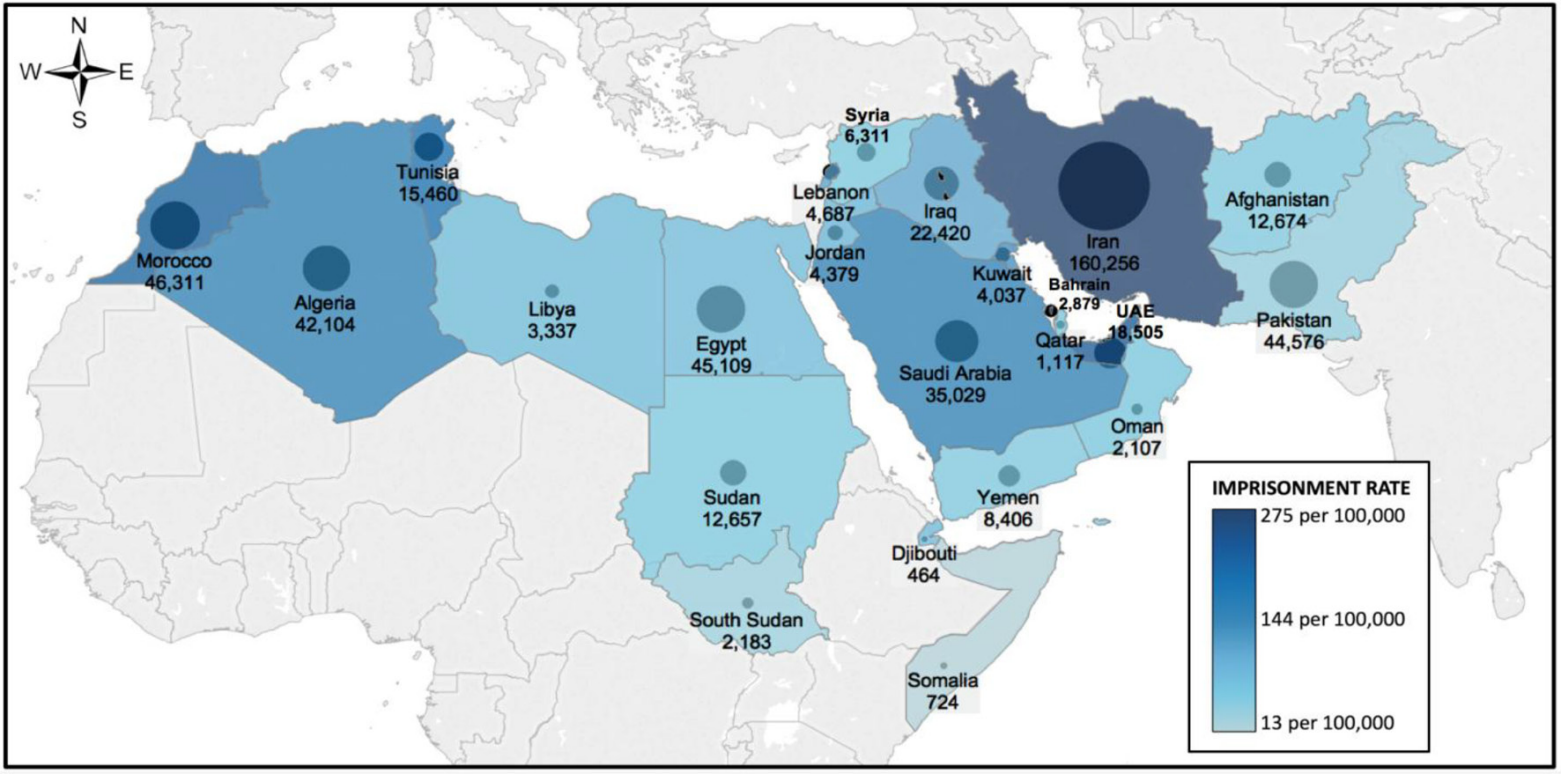
Imprisonment rate and prison population in countries of the Middle East and North Africa. Circles present a visual representation of the estimated size of the prison population. The number of prisoners is presented below the circle for each country. No data on imprisonment rate or prison population were available for Palestine.

We estimated that there are 496,000 prisoners across MENA (no prisoner estimate available for Palestine, therefore estimate is across 23 countries). As reported by the World Prison Studies [42], the vast majority of prisoners in MENA are male. The estimated proportion of females (as percentage of prison population) ranges from 1.6% Yemen to 14.6% in Qatar [42]. The average imprisonment rate is 114 per 100,000 adults (range: 33/100,000 in South Sudan to 284/100,000 in Iran; Figure 1). The prison population appears to be dynamic; it was reported in 2010 that there were as much as 200,000 prisoners in Iran in 245 prisons and detention centres, and 450,000 persons were estimated to enter and exit prisons annually [9]. In Afghanistan, it has been projected that the prison population will increase to 30,000 persons, mostly because of drug-related incarceration, by the end of 2015 [43].

#### Drug-related incarceration and drug use in prison

A strong link between drug use and incarceration has been documented in MENA. Across different studies, 45 [9], 48 [44], 60 [45], and 98.5% [46] of prison inmates in Iran, 58% in Sudan [47], and 68% prisoners in Syria [48] were incarcerated for drug-related offences. Similarly, 30.2% of PWID in Lebanon [49], and 61 to 83% of different male PWID populations in Oman [50] were incarcerated for drug-related offences. It was estimated that more than 400,000 people are arrested in Iran annually on drug-related charges [51], though a large proportion are not expected to serve long sentences, resulting in a high prisoner turnover [52].

In Iran and Sudan, 64% [44] and 26.1% [47] of prisoners reported a history of drug use. Continued use of drugs while in prison was reported by 67% of incarcerated drug users in Algeria [53]; 18.7% [54] and 91% [55] in Iran; 26 to 44% in Oman [50]; 15.1% [56] and 59.2% [57] in Pakistan; and 22% in Syria [58]. Similarly, history of previous incarceration was reported by over half of PWID [59] in Afghanistan; 40.4 [60], 41.0 [55], 75.3 [54], 76.2 [61], and 94.0% [62] in Iran; 45.9% in Morocco [63]; and 14.0 [64], 33 [65], 40.0 [64], and 84.0% [57] in Pakistan.

### Risk behaviours and incarceration

#### Injecting drug use and incarceration

Injecting drug use in prison appears to be present in several countries. Injecting drugs while incarcerated was reported by 17.4% of PWID in Afghanistan [59]; 23.2% in Egypt [66]; 6.6% [54], 27.6 to 53.6% [67], and 85% [55] in Iran; 19.8% in Morocco [63]; 5 to 11% in Oman [50]; and 11% in Syria [48]. Similarly, 2.2% of prisoners in Sudan [47] and 0.2% of prisoners (12% of whom were PWID) in Lebanon [68] injected drugs in prison. Several reports indicated initiation of drug injection in prisons. In Iran, for example, 5.7% of newly admitted prisoners and 21.3% of existing inmates started injecting while in prison [67].

Use of non-sterile injecting equipment in prisons has been reported by 30% of PWID in Afghanistan [69]; 6.1 [70], 12 [71], 16.8 [72], 27.1 [54], 36 [73], 47.3 [74], and 48.5% [61] in Iran; 7% in Lebanon [49]; 3 to 11% in Oman [50]; 46% in Pakistan [57]; and 80% in Syria [48]. In Sudan, 3.2% of prisoners reported use of non-sterile syringes while in prison [75].

#### Sexual risk behaviour and incarceration

High levels of sexual risk behaviour among prisoners have been documented both before and during imprisonment. Premarital and/or extramarital sex was reported by 11.4 [75], 41.9 [76], and 65.2% [47] of prisoners in Sudan and by 62.5% in Pakistan [56]. Among juveniles imprisoned in Pakistan, 76.4% reported sex before incarceration [77]. Sex with multiple partners was reported by 17.7% of prisoners in Sudan (>3 partners) (no timeframe specified) [75]. The average number of sexual partners was 5.2 (lifetime) among female prisoners in Morocco [78] and 16.7 among prisoners who were sexually active during the last year in Syria [58].

Sex with another male was reported by 1.6% of male prisoners in Iran [67], 19.3% in Pakistan [77], and 28.8% in Sudan [75]. In one study in Pakistan, 21.3% of male prisoners reported paying for sex with a male [57]. Sex with a female sex worker was reported by 21.6% and 28.5% of prisoners in two different settings in Afghanistan [43]; 11.9% in Iran [67]; 45.9% in Pakistan [57]; and 8.6% in Sudan [76]. In Syria, 21% of prisoners reported selling sex [58].

Overall, there was a history of low condom use among prisoners prior to incarceration. Only 4% [76] and 11.6% [75] of prisoners in Sudan ever used condoms. In Morocco, 9% of female prisoners reported using condoms [78], while in Syria, 10.5% of prisoners used a condom during last sex [57]. Condom use with casual partners was reported by 18.2% of prisoners in Pakistan [56] and 5.6% in Sudan [47]. In Iran, 16.7% of male prisoners used condoms with a male in the year prior to incarceration [67]. Condom use during commercial sex was reported by 27.3% of prisoners in Iran [67] and 24.7 [57] and 9.1% (with male sex workers) [57] in Pakistan.

There is evidence of sexual acts between males, mostly unprotected, happening in prisons. Among imprisoned male juveniles in Pakistan, 22.7% had sex in prison [77], whereas in Oman 6 to 18% of different male PWID populations reported doing so without condoms [50]. In Sudan, 31% of prisoners reported sex in prisons, and 28.8% reported this sex to be between males [75]. Anal sex with another male in prison was reported by 5.4% of male prisoners in Iran [53], 1.9 [79] and 2.6% [68] in Lebanon, and 1.4% in Sudan [47]. In Iran, 62.1% of prisoners did not use a condom during their last sexual contact in prison, and 18.5% reported having sex for money or drugs with at least two sexual partners [70]. Sexual abuse/forced sex by other prisoners has been also reported [44], by 10.3% of prisoners in Syria [58].

#### Tattooing and use of non-sterile razors and incarceration

Different studies reported a history of tattooing among prisoners, though few reported actual tattooing in prison. In Lebanon, 2.3% of prisoners had a tattoo done in prison [79], compared with 12.9% in Iran [52]. Also in Iran, 25.0 [67] and 40.6% [62] of imprisoned PWID reported being tattooed in prison. It was reported that 23.6 [61] and 31% [80] of prisoners in Iran, 26.7% in Pakistan [57], and 23.4% in Sudan shared or re-used shaving razors/blades in prison [75].

### Incarceration and risk of exposure to HIV and HCV infections

Risk behaviours in prison, such as injecting drugs, use of non-sterile injecting equipment, tattooing, and use of non-sterile toiletries, have been documented as modes of HIV and HCV exposure.

#### Incarceration as a risk factor for HIV and HCV

Largely because of injecting drug use, prior incarceration has been reported as a risk factor for both HIV and HCV in MENA, though most evidence originates from Iran. Here, imprisoned PWID with a history of previous incarceration had 2.9 to 4.0 [67] the odds of HIV infection compared with those entering prison for the first time, with higher OR observed with increased number of prior incarcerations [67].

Similarly, repeated incarceration was significantly associated with HCV infection among prisoners in two studies in Iran [81] and Lebanon [79] (analyses at bivariate level). After adjusting for other confounders, previous incarceration was significantly associated with HCV infection in three other studies from Iran. PWID in a mandatory detention centre had four times the odds of being HCV positive if they were previously incarcerated (OR of 4.4) [54]. Among imprisoned drug users, the OR of HCV infection was 3.1 and 6.7 for those who had 2 to 5 and >5 previous incarcerations, respectively, compared with new entrants [82]. Similarly, length of lifetime incarceration was significantly associated with HCV infection with ORs of 2.4 and 3.4 for those incarcerated for a total of 7 to 12 months and more than 12 months, respectively, compared with those who had never been incarcerated or been in prison for <6 months [83].

Imprisonment was also found to be a significant risk factor for HIV and HCV co-infection among incarcerated PWID in Iran (OR of 7.5) [84].

#### History of injecting risk behaviour among incarcerated populations as a risk factor for HIV and HCV infections

Different studies among incarcerated populations, but mostly from Iran, have linked injecting drug use to HIV or HCV exposure. In a male prison setting in Pakistan, injecting drug use was found to be a significant predictor of infection, irrespective of whether it was HIV, HCV, or hepatitis B virus (OR of 24.3) [57]. In Iran, history of drug injection was associated with 4.5 [85] and 7.8 [52] increased odds of HIV infection among prisoners, and injecting drugs in prison was found to be the greatest risk factor for HIV infection among incarcerated PWID (OR of 6.7) [67]. Similarly, history of using an opioid in jail in Iran was a risk factor for HIV infection (OR of 2.1) [86].

A number of studies of prisoners in different countries reported ORs of 4.1 [87], 8.1 [82], 14.7 [85], 24.7 (females) [88], and 134.4 (juvenile inmates) [81] for injecting drug use as a risk factor for HCV infection. Incarcerated prisoners who injected drugs had a significantly higher HCV prevalence compared with non-injecting prisoners (46.4% versus 2.0%) [56]. Age at first injection was found to be a significant risk factor for HCV among prisoners; first injection before the age of 25 resulted in 2.7 times the odds of infection [54].

#### History of use of non-sterile injecting equipment among incarcerated populations as a risk factor for HIV and HCV infections

Among incarcerated PWID in Iran, use of non-sterile syringes resulted in a significantly increased risk of HIV and HCV co-infection (OR of 4.5) [84]. Also in Iran, use of non-sterile needles was a significant risk factor for HIV in a prison setting (OR of 5.3) [67]. In Pakistan, history of use of non-sterile needles/syringes was the main risk factor for HIV infection among incarcerated PWID (OR of 2.0) [89].

#### History of tattooing or use of non-sterile razors as risk factors for HIV or HCV infections

Tattooing inside prison was associated with an increased risk of HIV infection (OR of 1.3 [62] and 1.8 [67] in Iran, and 2.8 in Egypt [87]) and HCV infection (OR of 2.0 in Iran) [83]. History of tattooing was found to be a significant risk factor for HCV infection in other studies in Iran including among prisoners (OR of 2.1 [52] and 100 [80]) and imprisoned PWID (OR of 1.8 [82], 2.3 [67], and 2.3 [54]).

Tattooing and use of non-sterile toiletries (brushes and razors) were found to be significant risk factors for HIV infection in an Egyptian prison (tattooing: OR of 2.8; toiletries: OR of 3.9) [87]. Use of non-sterile razors was found to be a significant predictor of HCV infection in a Pakistani prison (OR of 18.0) [56].

### HIV and HCV infections among prisoners

#### HIV outbreaks in MENA prisons and the HIV epidemic

Outbreaks of HIV in prisons have been documented globally [2] and prisons have played a role in the emergence of HIV epidemics in different countries [4,6]. The MENA region is no exception where there is evidence that outbreaks of HIV in prisons, specifically in Iran and Pakistan, were the catalysts of the large HIV epidemics seen among PWID.

The first HIV case among PWID in Iran was reported in 1992 [90]. Few cases were reported in the three following years [90]; however, starting from 1996, the number of reported cases suddenly rose 30-fold, which appeared to be linked to HIV outbreaks in prisons [90,91]. In Kermanshah prisons, the first HIV case was reported in 1995, followed by 58 cases in 1996 and increasing to 407 cases in 1997 to 1998 [92].

Following these prison HIV outbreaks, HIV prevalence started rising among PWID across Iran regardless of incarceration. By 2003, HIV prevalence reached concentration stage in most surveys (HIV prevalence >5%) [91]. Through overlapping risk behaviours [10,15,67,91,93–95], HIV was introduced to the sexual networks of MSM [15,96] and heterosexual commercial sex [94,97–99]. HIV transmission to spouses of PWID increased; the contribution of spouses to the number of notified HIV cases increased four-fold from 0.5% of all cases in 2001 to 2% in 2004 [91]. The vast majority of infected women in Iran acquired the infection from their (predominantly PWID) HIV-infected husbands [100–102].

Similar evolution of the HIV epidemic occurred in Pakistan. After years of virtually zero HIV prevalence among PWID [10,103–106], an HIV outbreak was identified among PWID in a prison in 2003. Immediately thereafter, HIV started a rapid growth among PWID across Pakistan regardless of incarceration [107–109]. In Karachi, for example, HIV prevalence in 2004 increased from less than 1 to 23% in less than six months [110] and reached 42% by 2011 [108]. Today, the HIV epidemic among PWID in Pakistan is one of the world's largest. Through overlapping risk behaviours, the epidemic among PWID appears to have ignited an HIV epidemic among MSM and transgender people [10,15,28]. Predictably, spouses of PWID were affected, with the majority of infected women acquiring HIV from their infected spouses [111].

#### HIV and HCV prevalence and incidence among prisoners in MENA

**Figure 2:**
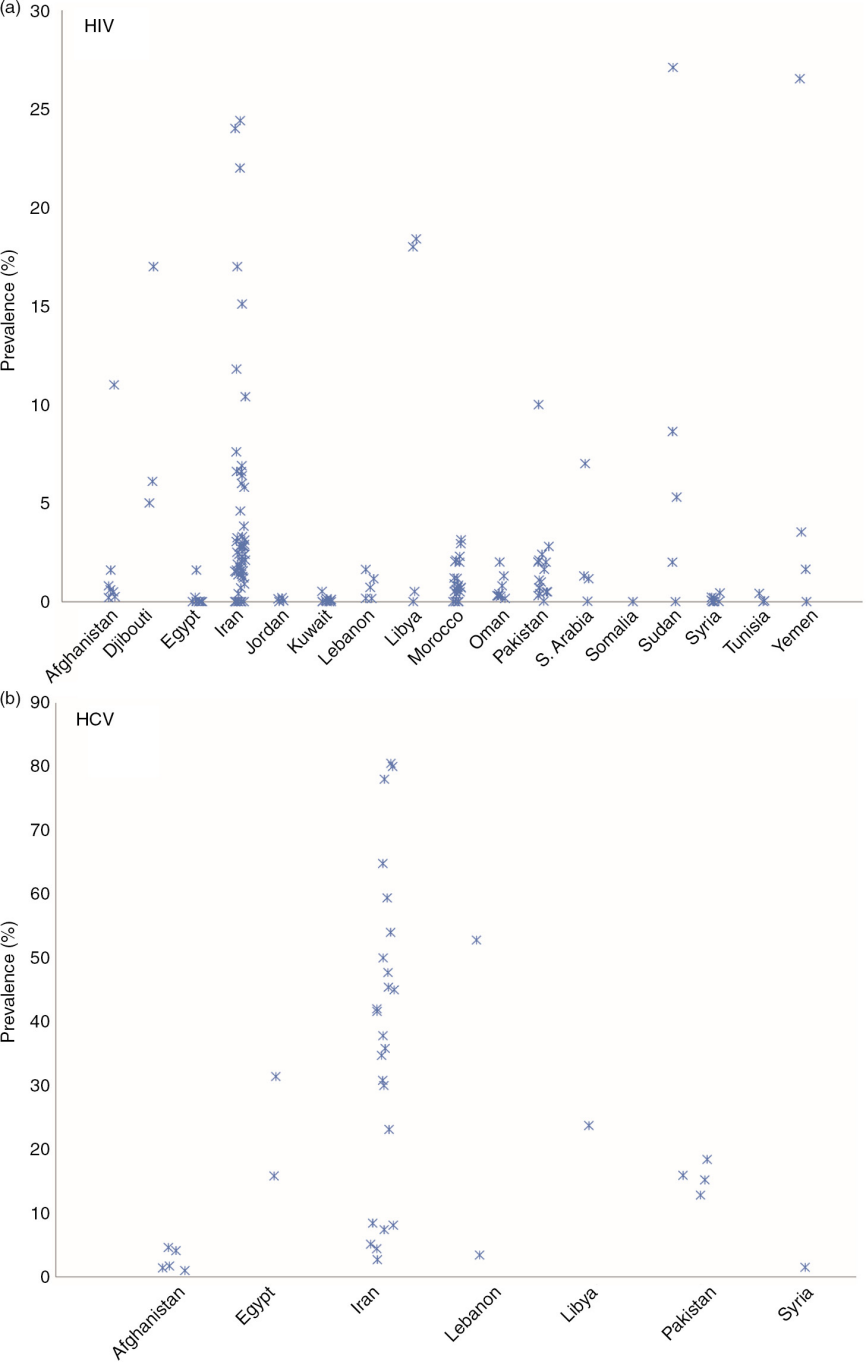
HIV and HCV prevalence among incarcerated populations in MENA countries. Three outliers (prevalence >40%; Iran (*n*=2) and Libya (*n*=1)) were removed from Figure 2a to improve display.

Data on HIV prevalence in prison were identified in 20 countries, with no data available in Algeria, Bahrain, Qatar, and UAE. Only one study, from Iran, provided data on HIV incidence, measuring an annualized incidence rate of 16.8% in a mandatory detention centre in Tehran [112]. Data on HCV prevalence in prison were identified in seven countries: Afghanistan, Egypt, Iran, Lebanon, Libya, Syria, and Pakistan. The countries that contributed the largest number of data points were Iran, Morocco, and Pakistan; and HIV and HCV prevalence data from these countries are listed in Table 1. HIV and HCV prevalence data from the remaining countries are listed in Table 2. HIV and HCV prevalence data among prisoners at the country-level are further summarized in Figure 2. As can be seen from the tables, the majority of prevalence data points referred to both males and females (*n*=71), with 36 prevalence data points specifically reporting on male prisoners and 22 data points specifically reporting on female prisoners.

**Table 1:** Prevalence of HIV and HCV in prisons in Iran, Morocco, and Pakistan

Country	Reference	Year of estimate	HIV prevalence (%)	HCV prevalence (%)	Sex	Drug-use status
Iran	Behrooz, 2011 [115]	Unknown	–	54	–	PWID
	Kaffashian, 2011 [116]	Unknown	–	42.0	–	PWID and non-PWID
	Afshar, 2005 [44]	Unknown	–	40–60	Both	Mostly PWID
	Nassirimanesh, 2002 [117]	Unknown	–	78	–	–
	Haghdoost, 2013 [92]	1991–2007	2.8	–	–	PWID and non-PWID
	Zali, 2001 [118]	1995	–	45.0	Males	PWID
	Rahimi-Movaghar, 2012 [119]	1998–2006	11.8	–	Both	PWID
	Amin-Esmaeili, 2012 [120]	1998–2007	0.4	–	Both	Non-PWID
	Shabazi, 2014 [121]	1999	1.56	–	Both	PWID and non-PWID
	Shabazi, 2014 [121]	2000	1.81		Both	PWID and non-PWID
	Zakizad, 2009 [80]	2001	–	30.8	Males	PWID and non-PWID
	Rowhani-Rahbar, 2004 [122]	2001	6.9	59.4	–	PWID
	UNAIDS, 2002 [123]	2001	12.0–63.0	–	–	PWID
	Khani, 2003 [71]	2001	1.2	47.7	Males	PWID and non-PWID
	Shabazi, 2014 [121]	2001	3.12	–	Both	PWID and non-PWID
	Mir-Nasseri, 2011 [84]	2001–2002	17.0	80.5	Both	PWID
	Davoodian, 2009 [124]	2002	15.1	64.8	–	PWID
	Jahani, 2005 [94]	2002	0	2.7	Females	PWID and non-PWID
	Shabazi, 2014 [121]	2002	3.83	–	Both	PWID and non-PWID
	Alizadeh, 2005 [72]	2002	0.9	30.0	Both	PWID and non-PWID
	Afshar, 2005 [44]	2002	2.1	–	Both	Mostly PWID
	Behnaz, 2007 [125]	2002–2003	5.8	23.1	Both	PWID and non-PWID
	Javadi, 2006 [73]	2003	–	35.8	Males	PWID and non-PWID
	Javadi, 2007 [126]	2003	6.6	37.8	Males	–
	Pourahmad, 2007 [85]	2003	6.4	34.7	Males	PWID and non-PWID
	Shabazi, 2014 [121]	2003	2.78	–	Both	PWID and non-PWID
	Afshar, 2005 [44]	2003	2.3	–	Both	Mostly PWID
	Farhoudi, 2003 [67]	2003	22.0	–	Males	PWID (newly arrived prisoner)
	Farhoudi, 2003 [67]	2003	6.0	–	Males	PWID (newly arrived, never incarcerated)
	Farhoudi, 2003 [67]	2003	24.0	–	Males	PWID (existing prisoner)
	Amiri, 2007 [82]	2003	–	45.4	Males	PWID and non-PWID
	Azarkar, 2007 [127]	2004	0	8.4	Males	PWID and non-PWID
	Azarkar, 2007 [127]	2004	0	5.1	Females	PWID and non-PWID
	Shabazi, 2014 [121]	2004	3.05	–	Both	PWID and non-PWID
	Afshar, 2005 [44]	2004	3.3	–	Both	Mostly PWID
	Ghannad, 2009 [128]	2004–2005	2.5	–	–	–
	Ghannad, 2009 [128]	2004–2007	2.4	–	–	–
	Shabazi, 2014 [121]	2005	3.24	–	Both	PWID and non-PWID
	Alavi, 2012 [129]	2005–2006	50.2	–	Males	PWID
	Alavi, 2012 [129]	2005–2006	10.4	–	Males	Inhalant drug users
	Alavi, 2012 [129]	2005–2006	7.6	–	Males	Oral drug users
	Ghannad, 2009 [129]	2005–2006	2.4	–	–	–
	Shabazi, 2014 [121]	2006	2.83	–	Both	PWID and non-PWID
	Kheirandish, 2009 [54]	2006	–	80.0	Males	PWID
	Kheirandish, 2010 [86]	2006	24.4	–	Males	PWID
	Ghanbarzadeh, 2006 [46]	2006	0	–	Females	PWID and non-PWID
	Ghannad, 2009 [128]	2006–2007	1.9	–	–	–
	Shabazi, 2014 [121]	2007	1.71	–	Both	PWID and non-PWID
	Kazerooni, 2010 [70]	2007	6.6	–	Males	PWID
	WHO, 2012 [130]	2007	1.5	–	–	–
	WHO, 2012 [130]	2008	2.8	–	–	–
	Shabazi, 2014 [121]	2008	2.01	–	Both	PWID and non-PWID
	Azarkar, 2010 [131]	2008	0	8.1	Both	PWID and non-PWID
	Nokhodian, 2012 [81]	2008–2009	–	4.4	Both	PWID and non-PWID
	WHO, 2012 [130]	2009	1.6	–	–	–
	Dibaj, 2013 [89]	2009	4.6	–	–	PWID
	Shabazi, 2014 [121]	2009	1.54	–	Both	PWID and non-PWID
	Navadeh, 2013 [52]	2009	2.1	–	Both	PWID and non-PWID
	Nokhodian, 2012 [88]	2009	0	7.4	Females	PWID and non-PWID
	Kassaian, 2012 [132]	2009	–	41.6	Both	PWID and non-PWID
	WHO, 2012 [130]	2010	1.4	–	–	–
	Shabazi, 2014 [121]	2010	1.37	–	Both	PWID and non-PWID
	WHO, 2012 [130]	2011	2.9	–	–	–
	Shabazi, 2014 [121]	2011	1.28	–	Both	PWID and non-PWID
	WHO, 2012 [130]	2011	0.7	–	–	–
	WHO, 2012 [130]	2012	1.6	–	–	–
Morocco	Elharti, 2001 [133]	2001	0.7	–	Males	–
	Elharti, 2001 [133]	2001	0	–	Females	–
	Khattabi, 2005 [134]	2001	2.3	–	Females	–
	Khattabi, 2005 [134]	2002	1.18	–	Males	–
	Khattabi, 2005 [134]	2002	0.72	–	Females	–
	Khattabi, 2005 [134]	2002	3.14	–	Females	–
	Ministry of Health, 2003–2004 [135]	2003	0.83	–	Males	–
	Khattabi, 2005 [134]	2003	0	–	Females	–
	Khattabi, 2005 [134]	2003	2.03	–	Females	–
	El Ghrari, 2007 [78]	2004	2.0	–	Females	PWID and non-PWID
	Khattabi, 2005 [134]	2004	1.2	–	Females	–
	Khattabi, 2005 [134]	2004	0.9	–	Females	–
	Khattabi, 2005 [134]	2004	0.61	–	Males	–
	Bennani, 2006 [136]	2005	0	–	Females	–
	Bennani, 2006 [136]	2005	0.61	–	Males	–
	Bennani, 2006 [136]	2005	2.94	–	Females	–
	Ministry of Health, 2010 [137]	2006	2.1	–	Females	–
	Ministry of Health, 2010 [137]	2006	0.53	–	Males	–
	Ministry of Health, 2010 [137]	2008	0.77	–	Males	–
	Ministry of Health, 2010 [137]	2008	0	–	Females	–
	Ministry of Health, 2010 [137]	2009	0.73	–	Females	–
	WHO, 2012 [130]	2009	0.4	–	–	–
	WHO, 2012 [130]	2010	0.5	–	–	–
Pakistan	Mujeeb, 1993 [138]	Unknown	1.64	–	Both	–
	Butt, 2010 [56]	Unknown	–	18.4	Males	PWID and non-PWID
	Baqi, 1998 [103]	1993–1994	0.03	–	Males	PWID and non-PWID
	Baqi, 1998 [103]	1993–1994	1.1	–	Females	Non-PWID
	Safdar, 2009 [139]	2006	2.8	–	Both	–
	Safdar, 2009 [139]	2006	0.7	–	Males	–
	Safdar, 2009 [139]	2006	10.0	–	Females	–
	Safdar, 2009 [139]	2006	0.5	–	–	–
	Safdar, 2009 [139]	2006	0.4	–	–	–
	Safdar, 2009 [139]	2006	0.5	–	–	–
	Safdar, 2009 [139]	2006	0.3	–	–	–
	Safdar, 2009 [139]	2006	2.1	–	–	–
	Safdar, 2009 [139]	2006	0.7	–	–	–
Pakistan	Safdar, 2009 [139]	2006	1.0	–	–	–
	Kazi, 2010 [57]	2007–2008	2.0	15.2	Males	PWID and non-PWID
	Gorar, 2010 [140]	2008–2009	–	12.8	–	–
	Shah, 2013 [141]	2009	2.4	–	Males	–
	Nafees, 2011 [142]	2009	2.01	–	Both	–
	Anwar, 2011 [143]	2009	–	15.9	Both	–

PWID, people who inject drugs.

“–” represents not reported or unclear in the original data source.

**Table 2:** Prevalence of HIV and HCV among incarcerated populations in MENA countries, excluding Iran, Morocco, and Pakistan

Country^a^	Reference	Year of estimate	HIV prevalence (%)	HCV prevalence (%)	Sex	Drug-use status
Afghanistan	World Bank, 2008 [144]	2008	11.0	–	Male	PWID
	National AIDS Control Program, 2010 [145]	2009	0.6	1.7	–	–
	National AIDS Control Program, 2010 [145]	2009	1.6	4.1	–	–
	WHO, 2013 [146]	2011	0.25	0.98	–	–
	WHO, 2012 [130]	2011	0.2	–	–	–
	WHO, 2012 [130]	2012	0.5	4.6	–	–
	WHO, 2012 [130]	2012	0.8	1.4	–	–
Djibouti	Shresta, 1999 [147]	1993	6.1	–	Both	–
	Shresta, 1999 [147]	1993	5.0	–	Male	–
	Shresta, 1999 [147]	1993	17.0	–	Females	–
Egypt	El-Ghazzawi, 1987 [148]	Unknown	0	–	Both	–
	Mohamed, 2013 [87]	Unknown	0	15.8	Both	PWID and non-PWID
	Ministry of Health, 2001 [149]	1986–2001	0.01	–	–	–
	HIV/AIDS Surveillance database (US Census Bureau), 2013 [150]	1991–1999	0	–	Both	–
	Quinti, 1995 [151]	1992–1994	1.6	31.4	–	–
	Murugasampillay, 1995 [152]	1993	0	–	Both	–
	HIV/AIDS Surveillance database (US Census Bureau), 2013 [150]	2004	0.02	–	Both	–
	WHO, 2012 [130]	2008	0.22	–		
	WHO, 2012 [130]	2008	2.8	–	–	–
	WHO, 2012 [130]	2009	1.6	–	–	–
	WHO, 2012 [130]	2010	1.4	–	–	–
	WHO, 2012 [130]	2011	0.7	–	–	–
	WHO, 2012 [130]	2012	1.6	–	–	–
Iraq	Shrestha, 1999 [147]	1993–1999	0	–	–	–
	WHO, 2012 [130]	2010	0	–	–	–
Jordan	El-Tayeb, 1995 [150]	1987	0.14	–	Both	–
	El-Tayeb, 1995 [150]	1990	0.05	–	Both	–
	El-Tayeb, 1995 [150]	1991	0.2	–	Both	–
	WHO, 2012 [130]	2007–2010	0	–	–	–
Kuwait	Kuwait National AIDS program [150]	1984–1998	0.05	–	–	–
	Shreshta, 1999 [147]	1993	0.14	–	Both	–
	Shreshta, 1999 [147]	1994	0.11	–	Both	–
	Shreshta, 1999 [147]	1995	0	–	Both	–
	Shreshta, 1999 [147]	1996	0	–	Both	–
	Shreshta, 1999 [147]	1997	0.09	–	Both	–
	Shreshta, 1999 [147]	1998	0	–	Both	–
	Shreshta, 1999 [147]	1999	0	–	Both	–
	UNAIDS, 2004 [153]	1999	0.52	–		–
	UNAIDS, 2004 [153]	2000	0	–		–
	WHO, 2012 [130]	2011	0.11	–		–
Lebanon	Shreshta, 1999 [147]	1993	1.16	–	Both	–
	Shreshta, 1999 [147]	1994	1.63	–	Both	–
	Shreshta, 1999 [147]	1995	0.72	–	Both	–
	National AIDS Control Programme [154]	2007–2008	–	52.8	–	PWID and non-PWID
	Mishwar, 2008 [68]	2007–2008	0.16	–	Males	PWID and non-PWID
	Mahfoud, 2010 [79]	2007–2008	0.17	3.4	Males	PWID and non-PWID
Libya	Sammud, 2005 [155]	Unknown	18.0	–	–	–
	Shazly, 1991 [156]	1990	0	–	Both	–
	Shazly, 1991 [156]	1990	0.51	–	Both	–
	Dolan, 2007 [2]	2003	60.0	–	–	PWID
	Ziglam, 2012 [157]	2006	18.4	23.7	Males	–
Oman	Shreshta, 1999 [147]	1996	2.01	–	Both	*–*
	Shreshta, 1999 [147]	1997	1.3	–	Both	*–*
	Shreshta, 1999 [147]	1998	0.28	–	Both	*–*
	Shreshta, 1999 [147]	1999	0.17	–	Both	*–*
	WHO, 2012 [130]	2007	0.4	–		–
	WHO, 2012 [130]	2008	0.3	–	–	–
	WHO, 2012 [130]	2009	0.8	–	–	–
	WHO, 2012 [130]	2010	0.3	–	–	–
	WHO, 2012 [130]	2011	0.3	–	–	–
Palestine	WHO, 2012 [130]	2010	0	–	–	–
Saudi Arabia	Madani, 2004 [158]	1984–2001	7.0	–	–	–
	WHO, 2012 [130]	2010	0.02	–	–	–
	WHO, 2012 [130]	2011	1.3	–	–	–
	Ministry of Health, 2014 [159]	2012	1.16	–	–	–
Somalia	Ahmed, 1997 [160]	1997	0	–	Males	–
Sudan/South	Burans, 1990 [161]	1988 (?)	0	–	–	–
Sudan	UNAIDS, 2004 [162]	2002	2.0	–	–	–
	Assal, 2006 [75]	2005	5.3	–	Males	–
	Assal, 2006 [75]	2005	8.63	–	Both	–
	Assal, 2006 [75]	2005	27.1	–	Females	–
Syria	Shrestha, 1999 [147]	1993	0.07	–	Both	–
	Shrestha, 1999 [147]	1994	0.44	–	Both	–
	Shrestha, 1999 [147]	1995	0	–	Both	–
	Shrestha, 1999 [147]	1996	0.2	–	Both	–
	Shrestha, 1999 [147]	1997	0	–	Both	–
	Shrestha, 1999 [147]	1998	0	–	Both	–
	National Progress report on AIDS, 2012 [163]	2011	0.22	–	–	–
	Ministry of Health, 2014 [58]	2013–2014	0	1.5	Both	–
Tunisia	Gharbi, 1988 [164]	1987 (?)	0.42	–	Both	–
	Ministry of Health, 1990 [165]	1988–1989	0.05	–	Both	
	Shrestha, 1999 [147]	1994–1999	0	–	Both	–
Yemen	Shrestha, 1999 [147]	1993–1994	0	–	Both	–
	Shrestha, 1999 [147]	1995	3.53	–	Both	–
	Shrestha, 1999 [147]	1996	1.64	–	Both	–
	Shrestha, 1999 [147]	1997	44.43	–	Both	–
	Shrestha, 1999 [147]	1998	26.53	–	Both	–

a No data available for Algeria, Bahrain, Qatar, and UAE.

PWID, people who inject drugs.

“–” represents not reported or unclear in the original data source.

There was considerable variation in HIV and HCV prevalence among incarcerated populations, both within and between MENA countries (Tables 1 and 2, Figure 2). The median HIV prevalence among incarcerated populations was 0.6% in Afghanistan (range: 0.2–11.0), 6.1% in Djibouti (range: 5.0–17.0), 0.01% in Egypt (range: 0–1.6), 0.1% in Jordan (range: 0–0.1), 2.5% in Iran (range: 0–63.0), 0.05% in Kuwait (range: 0–0.5), 0.7% in Lebanon (range: 0.2–1.6), 18.0% in Libya (range: 0–60.0), 0.7% in Morocco (range: 0–3.1), 0.3% in Oman (range: 0.17–2.0), 1.1% in Pakistan (range: 0.03–10.0), 1.2% in Saudi Arabia (range: 0.02–7.0), 5.3% in Sudan and South Sudan (range: 0–27.1), 0.04% in Syria (range: 0–0.4), 0.05% in Tunisia (range: 0–0.4), and 3.5% in Yemen (range: 0–44.4).

The median HCV prevalence among incarcerated populations was 1.7% in Afghanistan (range: 1.0–4.6), 23.6% in Egypt (range: 15.8–31.4), 28.1% in Lebanon (range: 3.4–52.8), 15.6% in Pakistan (range: 12.8–18.4), 37.8% in Iran (range: 2.7–80.5), 1.5% in Syria, and 23.7% in Libya.

Across all countries, the median HIV prevalence was 0.9% (IQR: 0.1–2.8) and the median HCV prevalence was 23.7% (IQR: 6.3–45.2).

### Synthesis and triangulation of epidemiologic evidence

**Figure 3:**
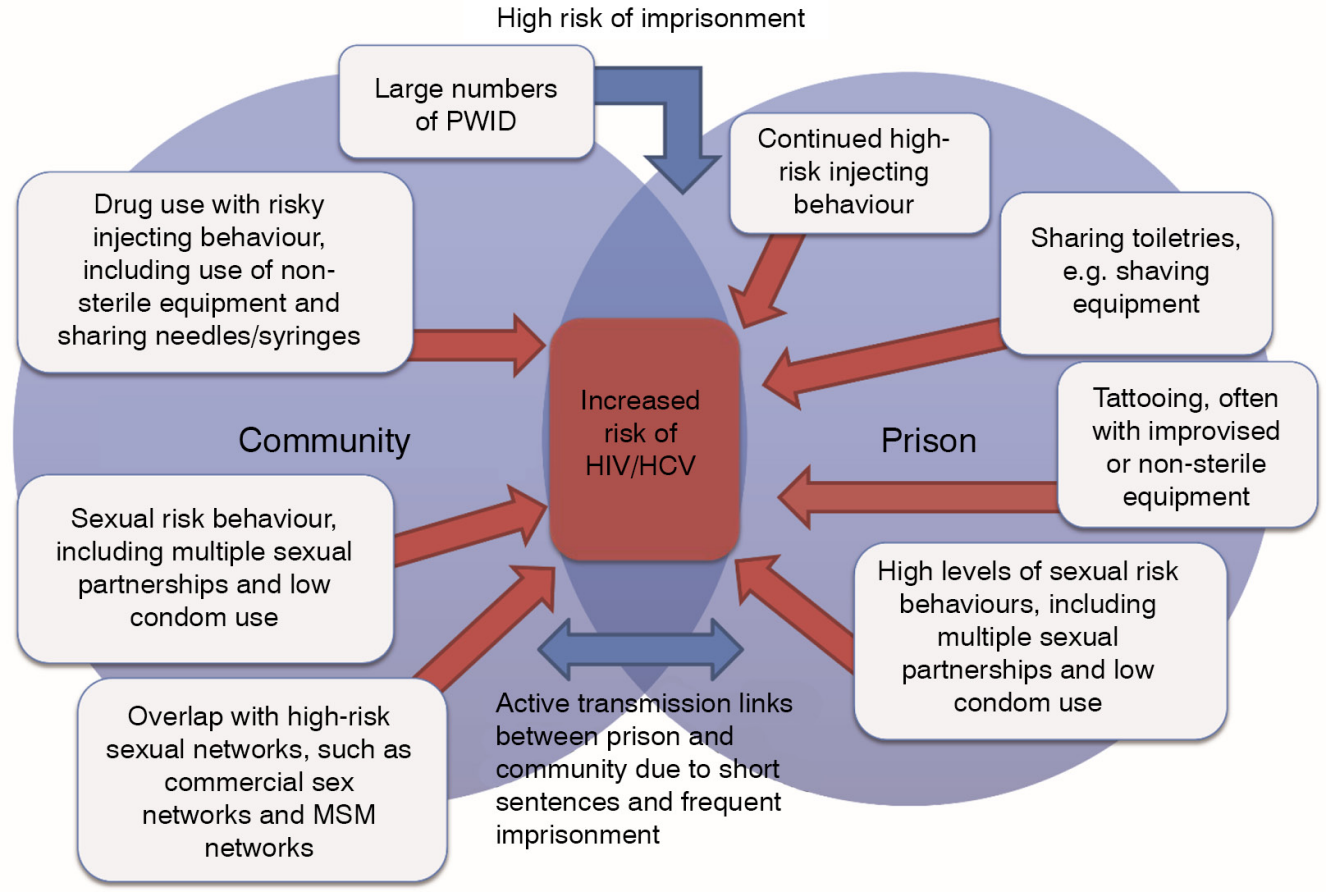
A schematic diagram of the dynamics of HIV and HCV infections and incarceration based on synthesis and triangulation of the epidemiologic evidence in the Middle East and North Africa. PWID, people who inject drugs; HCV, Hepatitis C virus; MSM, men who have sex with men.

Figure 3 shows a schematic illustration of the synthesis and triangulation of the epidemiologic evidence for HIV and HCV and incarceration in MENA. There is a large incarcerated population of about half a million prisoners in this region, with drug use often the cause of incarceration. Prisoners engage in risk behaviours that expose them to HIV and HCV. These behaviours, which are often initiated before imprisonment, are continued in prison and have been linked to HIV and HCV exposures during incarceration. Among these behaviours are use of non-sterile injecting equipment, drug use, multiple sexual partnerships and unprotected sex, and tattooing and use of non-sterile razors.

Incarceration in MENA has been documented to be an independent risk factor for HIV and HCV infections. These infections have been shown to have a much higher prevalence among incarcerated populations (Tables 1 and 2) than among the general population in this region [11,23]. With the usually short sentences for drug use and repeated imprisonments, there are active infection transmission links between prisons and community. These links have facilitated the spread of HIV and HCV to wider at-risk populations, such as when HIV outbreaks have occurred in prisons. Indeed, HIV outbreaks in the prisons of Iran and Pakistan have played a critical role in igniting large-scale HIV epidemics among PWID in different parts of these two countries. Despite the progress in understanding the dynamics of HIV and HCV and imprisonment in several countries, the situation remains poorly understood in over half of MENA countries because of limited epidemiologic evidence.

## Discussion

Prisons play an important role in the dynamics of HIV and HCV infections in MENA and have facilitated the emergence of large HIV epidemics in at least two countries. Incarceration is a main risk factor for HIV and HCV in this region. The prison environment is conducive to different forms of risk behaviour that expose prisoners to HIV and HCV; and these have been documented to be prevalent in MENA. The most important of these behaviours is injecting drug use. Drugs appear to be accessible in MENA prisons, though at a higher cost. In Iran, drugs were found to be 5 to 8 times more expensive in prisons than outside [113], which has been suggested as a reason for initiation of drug injection, as taking drugs through injection is more cost-effective [113,114]. The scarcity of raw opium and difficulty in hiding smoking are other factors promoting drug injection in prisons [54,55]. The in and out of prison transmission links appear to contribute significantly to HIV and HCV transmission in the wider at-risk populations in at least few countries. With the currently emerging HIV epidemics among PWID and MSM in different MENA countries, some of which have already reached substantial levels [10,15,28], prisons may play a growing role in the dynamics of infection transmission.

We estimated that there are approximately half a million prisoners in MENA. Prison populations vary considerably, with the regional average of 114 prisoners per 100,000 adults falling just below the global average of 144 per 100,000 [39]. Most of the prisoners in the MENA region are males [42], and the majority of the studies included in this review were conducted either among males or both sexes. Some countries did provide data on infection prevalence among female prisoners, such as Morocco [27], but it must be noted that even the World Prison Study report provided estimates of female prisoners in only 12 MENA countries [42].

A substantial proportion of these prisoners have been convicted of drug-related offences. The high HCV prevalence among prisoners, with a median of 24% across countries (Tables 1 and 2), further indicates that a large fraction of prisoners inject or injected drugs in the past. In a context of over 600,000 PWID in MENA [10], injecting drug use poses a major challenge. PWID continue to injected while incarcerated, often using non-sterile needles/syringes. While it appears to be the main mode of HIV/HCV exposure among prisoners, considerable sexual risk behaviour is also reported, as well as tattooing and use of non-sterile razors/toiletries. These may also contribute to HIV and HCV exposures.

The convergence of vulnerability and risks in prison is not unique to the region; similar situations have been reported in both developing [87,121,166] and developed countries [167–169]. HIV and HCV prevalence levels among incarcerated populations in MENA are also broadly consistent with those found globally [1–3]. Prisoners often practice high injecting and sexual risk behaviours even before incarceration; and the majority of prisoners come from vulnerable strata of society suffering from poorer health and fewer opportunities [169].

There is generally poor access in MENA to services that can reduce prisoners’ risk of infection exposure. Iran has made significant progress in implementing and expanding harm-reduction services in prisons, including needle exchange and methadone maintenance programmes [112]. Morocco has made plans for methadone replacement therapy in prisons [170]. Conjugal visiting rooms have been provided in some prisons in Iran and Sudan [75,91], although in Sudan the right to conjugal visits is rarely practiced [75]. The progress in the public health response in few countries, though modest, serves as an example of what could be feasible in other countries to enhance provision of sterile injecting equipment, methadone replacement therapy, HIV antiretroviral therapy, HCV treatment, condoms, and information to prevent HIV and HCV infections.

HIV and HCV voluntary counselling and testing needs to be made available to incarcerated populations along with harm reduction and treatment services. These services should be integrated within the wider scope of public health services for PWID, MSM, and commercial sex workers. Initiation of prevention and treatment services in prisons may provide an opportunity for expanding these services outside prisons. Prisons present a unique entry point for intervention, because many of the incarcerated individuals are part of the most at-risk “hidden” populations. Prisoners, upon release, could be enlisted to serve as interpersonal communication agents within their communities. Lastly, reduction in prison populations and less reliance on incarceration as a punishment should be considered.

Our study has several limitations. The data synthesized in this review were not extracted strictly through a specific systematic search of data on HIV/HCV in prisons. However, it is unlikely that we have missed consequential evidence that would affect our results and findings. The data were identified through broad and comprehensive systematic searches of different aspects of HIV and HCV epidemiology in MENA through two large-scale projects; in fact, the most comprehensive epidemiology projects for both infections in this region to date. It is possible that a systematic search of HIV/HCV in prisons may not have yielded as much data, as most data synthesized here were extracted from studies not focused on prisoners, but on other populations such as PWID. These studies could have been missed in a systematic search focusing strictly on HIV/HCV in prisons.

Nearly all included studies were cross sectional, making it difficult to discern temporal patterns and direct evidence of HIV/HCV incidence within prisons. The availability of data varied considerably between countries. A large fraction of data originated from only three countries: Iran, Morocco, and Pakistan. Hardly any meaningful data were identified for few countries. Though some studies reported prevalence data from a range of sites, many were conducted in a small number of prisons; these results may not be generalizable to the wider prison population of that country. Included studies may also suffer from limitations and biases; and the reporting quality varied across studies. The illegal and sensitive nature of risk behaviours, as well as social desirability, may have biased reported results in included studies, especially those with questions pertaining to sexual behaviour in an all-male environment. A number of studies highlighted the exclusion of questions about sexual behaviour in prison as a limitation of their data collection [58,72,79,82,87,142].

Our results show considerable heterogeneity, both in terms of risk behaviours and prevalence of HIV/HCV infections. This heterogeneity is likely to reflect true differences in the specific composition of the incarcerated populations from one setting to another, within or across countries. The representation of specific at-risk populations such as PWID or FSW can vary immensely from one setting to another; Iran, for instance, is much more affected by injecting drug use than Morocco [10].

## Conclusions

HIV and HCV prevalence among incarcerated populations in MENA vary considerably, but the risk factors for infection are present throughout the region. Prisons have played an important role in HIV and HCV dynamics and have facilitated the emergence of large HIV epidemics in Iran and Pakistan. Prisons could be playing a disproportionately larger role in HIV dynamics in MENA than elsewhere, as highlighted recently by UNAIDS [7]. The need to ensure access to HIV combination prevention services for at least 90% of prisoners by 2020 has been emphasized in the new UNAIDS strategy for 2016 to 2021 [7]. Despite this global push for improved services in prisons, regional response continues to lag needs. It is critical to expand access to harm reduction and treatment services, as well as HIV and HCV treatment. Increased and expanded HIV and HCV surveillance in prisons is also essential, for example through integrated bio-behavioural surveys. The status of these infections in prisons in several MENA countries continues to be poorly understood. By implementing prevention and care activities, and carefully monitoring these infections in prisons, these institutions could play a pivotal role in the control of, and eventual reduction in, HIV and HCV transmission well beyond prison settings.
